# Relationship Between Breakfast Skipping and Body Composition, Nutritional Status or Chronotype in Female University Students With Normal Body Weight

**DOI:** 10.33549/physiolres.935652

**Published:** 2025-12-01

**Authors:** Kohei SEIKE, Kazushige OSHITA, Yujiro ISHIHARA, Ryota MYOTSUZONO, Ken NAGAMINE

**Affiliations:** 1 Department of Sport Science, Kyushu Kyoritsu University, Japan; 2 Department of Human Information Engineering, Okayama Prefectural University, Japan; 3 Center for Fundamental Education, Okayama University of Science, Japan

**Keywords:** Breakfast, Normal weight obesity, Body fat, Muscle mass, Energy intake, Morningness-Eveningness Questionnaire

## Abstract

Breakfast, the first meal of the day, plays a critical role in energy balance and dietary regulation. Although normal weight-obesity (NW-O) is considered a body composition problem among female university students, whether breakfast skipping is associated with NW-O remains unknown. This study aimed to investigate the relationship between breakfast skipping and body composition, nutritional status, and chronotype in female university students with normal body mass index (BMI). Two hundred Japanese university students were divided into two groups: those who rarely ate breakfast (SKIP) and those who ate breakfast almost daily (TAKE). Body composition such as percentage of body fat (%BF), fat-free mass (FFM) and appendicular muscle mass (AMM), chronotype (Morningness-Eveningness Questionnaire; MEQ), dietary intake, and physical activity level (PAL) were compared between the two groups. Our results showed that PAL was not significantly different between the two groups. Although body weight and BMI were not significantly different between the two groups, %BF was significantly higher and FFM was significantly lower in the SKIP group than in the TAKE group. The SKIP group had a significantly higher proportion of body fat of >30 % (55.8 %) than the TAKE group (34.1 %). Although the total energy intake was not significantly different between the two groups, the percentage of fat intake was significantly higher in the SKIP group. The multiple regression analyses of all participants showed that %BF and AMM were negatively and positively associated with PAL and breakfast consumption frequency, respectively. The SKIP group had a significantly lower MEQ score and a significantly higher number of evening types (41.9 %) than the TAKE group (23.5 %). These results suggest that the habit of breakfast consumption and chronotype-specific lifestyle interventions are necessary to prevent NW-O.

## Introduction

University age is considered to be an appropriate period for transitioning to an autonomous life [1], and students are expected to develop appropriate lifestyle habits. However, university students are at risk of adopting unhealthy lifestyles such as a decrease in physical activity compared with previous years, which has been reported to lead to an increase in body weight or body mass index (BMI) [2,3]. Focusing on changes in body composition, a high percentage of body fat (%BF) despite a BMI within the normal range is referred to as “normal weight-obesity” (NW-O), which has a high risk of cardiometabolic dysregulation or systemic inflammation despite normal body weight [4–6]. Even if body weight is normal, a high %BF indicates that fat-free mass (FFM), such as muscle mass, is low, resulting in a state of low muscle mass and obesity. A certain prevalence of NW-O has been observed among female university students [7–9], and several studies have shown that the prevalence is higher among women than men [4,10]. Furthermore, the prevalence of NW-O in women increases with age [10], making preventive measures important for the younger generation. Therefore, identifying factors associated with NW-O in female university students is important for the prevention of lifestyle-related diseases and sarcopenia.

Physical activity and nutritional status have been reported to be associated with body composition in female university students [11–13]. Other factors such as eating behavior, particularly skipping breakfast, may also affect body composition. Breakfast, the first meal of the day, plays a critical role in energy balance and dietary regulation [14]. The National Health and Nutrition Survey in Japan 2019 showed that the percentage of skipping breakfast was high in women in their twenties [15]. Another survey of a large university population from 28 countries reported that 49.5 % of women skipped breakfast (sometimes, rarely, or never having breakfast) [16]. Several studies have suggested an association among skipping breakfast, obesity, and metabolic syndrome [17–19]. Moreover, a positive association between breakfast consumption frequency and muscle mass index has been reported in university students [11,20]. Additionally, skipping breakfast has been suggested to interact with individual morning and evening preferences [21,22]. Individuals have a time orientation called the chronotype, and those aged approximately 20 years, including university students, exhibit the latest chronotype, that is, they become evening types [23]. A previous study suggested that female university students with chronotype lateness and low physical activity have a body composition imbalance resulting in higher body fat and lower muscle mass [24].

As mentioned previously, NW-O is considered a body composition problem among female university students. However, whether skipping breakfast is associated with NW-O in female university students is unknown. Therefore, we conducted a cross-sectional survey of female university students with normal-range BMI to clarify the relationship between breakfast skipping and body composition, nutritional status, and chronotype.

## Methods

### Participants (Fig. 1)

The surveys and body composition measurements described below were conducted between late September and early December, in the morning (9–12 a.m.). To minimize the potential impact of seasonal variations on body composition measurements obtained using the bioelectrical impedance method [25], the measurement period was restricted to approximately two-and-a-half months. Participants were recruited from one junior college and two universities in different Japanese prefectures, and academic years were randomly chosen.

This study included 265 female Japanese university students who answered all questions in the questionnaire described below. According to the Japanese Guidelines for the Management of Obesity Disease [26], 43 students with BMI outside the normal range (<18.5 and ≥25.0) were excluded from the study, as the present study was conducted in the context of NW-O. Similar to a previous study [24], 22 students with extremely high physical activity level (PAL; ≥2.4) were excluded from this study, and data from 200 participants were included in the analysis. Based on the breakfast consumption frequency survey (described later), participants who reported eating breakfast almost daily were classified into the TAKE group (132 participants) and those who ate breakfast <2 d a week were classified into the SKIP group (43 participants). For ethical reasons, the participants were informed in advance that the survey would be anonymous, that it would be used for the purpose of this study and not for any other purpose, and that any data not used in the study would be discarded. If the results of the survey were to be published, the participants were informed that the collected data would be statistically processed and then published in a manner such that individuals could not be identified and that the survey would only be performed if participants consented. This study was approved by the Research Ethics Committee of Kyushu Kyoritsu University (approval number: 2022–08).

### PAL

The average daily activity and exercise time on weekdays during the previous month were assessed using a daily activity diary. The PAL of each activity was assessed by calculating the daily average of each classified activity using the product of the energy expenditure index, which was expressed as a multiple of the basal metabolic rate and activity time. This method was based on a study that estimated PAL according to the lifestyle of university students based on the “Dietary Reference Intakes for Japanese people” [12].

### Nutritional intake status and frequency of having breakfast

Nutritional intake status was evaluated using a food frequency questionnaire based on food groups (FFQg) (FFQ-NEXT short version; Kenpakusha, Japan) [27]. Participants were asked to report on their usual diet over the past year. The FFQg results were analyzed using the Microsoft Excel add-in software Eiyou Plus (Kenpakusha, Japan) to calculate the daily intake of total energy (EI), protein (PI), fat (FI), and carbohydrates (CI). The percentage of each macronutrient intake relative to EI was also calculated (%PI, %FI, and %CI, respectively).

In addition to the FFQg, frequency of breakfast consumption was assessed. In response to the question “How many days a week do you eat breakfast?,” respondents were asked to choose one of the following options “I do not eat breakfast” (Never), “I eat breakfast 1 or 2 d” (Rarely), “I eat breakfast 3 or 4 d” (Some days), or “I eat breakfast almost every day (≥ 5 d)” (Every Day).

### Morningness-Eveningness Questionnaire (MEQ) and Chronotype

The Japanese version of the MEQ [28,29] was used to assess the chronotypes. The questionnaire consisted of 19 questions that were scored (16–86) based on the answers. Individuals exhibiting a chronotype were classified as definitely morning type (MT; score: 70–86) or moderately MT (score: 59–69), neither type (NT; score: 42–58), moderately evening type (ET; score: 31–41), or definitely ET (score: 16–30) [28]. In this study, definitively MT and moderately MT were classified as MT, and definitively ET and moderately ET as ET.

### Body physique and composition

Body weight and composition were measured using a standing eight-electrode multi-frequency BIA body composition analyzer (MC-780A-N, Tanita, Japan). Body composition was estimated from the impedance values of three different alternating currents (5 kHz, 50 kHz, and 250 kHz) of ≤90 *μ*A applied through electrodes on the palms and plantar feet. All measurements were performed in the morning. Participants were asked to urinate and defecate before the measurements and not immediately after eating. Participants wiped their palms and plantar surfaces with alcohol-free wet wipes to clean and moisten them. The participants then stepped onto the electrode surface of the analyzer and grasped the hand electrodes to measure their body weight and composition. Body weight (kg) was divided by the square of height (m) to obtain the BMI. Additionally, %BF, FFM, appendicular muscle mass (AMM), and AMM/BMI were analyzed from the measured body composition.

### Statistical analyses

The mean and standard deviation (SD) of each variable were calculated for all participants. The participants were divided into two groups according to the frequency of breakfast consumption: the TAKE group, those who answered that they ate breakfast ‘Every day’, and the SKIP group, those who answered that they ‘rarely’ or ‘never’ ate breakfast. The Mann-Whitney *U* test was used to compare variables between the groups, and the d-value was calculated as the effect size using Cohen’s method. The proportions of participants with ET, NT, and MT in each group were compared using chi-squared tests. A chi-squared test was used to compare the proportion of participants in each group with %BF <30 % and ≥30 %. Although the criteria for %BF to assess NW-O vary and are not standardized, many studies have used a %BF of 30 % in women [6]. Further, a forced entry multiple regression analysis was performed to identify factors associated with %BF or AMM for all participants, including those who answered, ‘Some days’. The independent variables included age, PAL, MEQ score, nutrient intakes and breakfast consumption frequency (four levels; 1 = Never, 2 = Rarely, 3 = Some days, and 4 = Every day). Nutrient intake was modelled using either EI alone (model 1) or the three macronutrients (model 2).

The StatFlex statistical software (ver. 7.0.10; Artec, Osaka, Japan) was used for performing the aforementioned statistical procedures, and the statistical significance level set at *P* < 0.05. Effect sizes were graded as *d* < 0.2 trivial effect, *d* = 0.2–0.5 small effect, *d* = 0.5–0.8 moderate effect, and 0.8 < *d* large effect [30].

## Results

Table 1 shows the means and SDs of all participants in the SKIP and TAKE groups. Regarding body physique and composition, %BF was significantly lower and FFM, AMM, and AMM/BMI were significantly higher in the TAKE group than in the SKIP group, and these effect sizes were moderate. The MEQ scores were significantly lower in the SKIP group than in the TAKE group, and the effect sizes were moderate. The PAL was not significantly different between the two groups, and the effect size was small. Although the EI was not significantly different between the two groups, with a small effect size, the % FI was significantly lower in the TAKE group.

The number of participants with % BF ≥30 % and <30 % were 74 (37.0 %) and 126 (63.0 %), respectively, among all participants. Although there were significantly fewer participants with %BF <30 % (44.2 %) and significantly more with %BF ≥30 % (55.8 %) in the SKIP group, there were significantly more participants with %BF <30 % (65.9 %) and significantly fewer with %BF ≥30 % (37.0 %) in the TAKE group %) (Table 2).

The chronotypes of all participants were as follows: 19 (9.5 %) were MT, 121 (60.5 %) were NT, and 60 (30.0 %) were ET. The chronotype classifications of the SKIP and TAKE groups are shown in Table 3. Although the number of MT and NT was not significantly different between the two groups, the number of ET was significantly higher in the SKIP group than in the TAKE group.

Table 4 shows the results of multiple regression analysis for all participants. The multiple regression analysis predicting %BF using nutrient intake as EI (model 1) was statistically significant (*F* (5, 194) = 12.04, *P* < 0.01), explaining approximately 22 % of the variance (adjusted R^2^ = 0.22). Among the independent variables, PAL (standardized *β* = −0.42, *P* < 0.01) and breakfast consumption frequency (standardized *β* = −0.14, *P* = 0.02) were significantly associated with %BF. The multiple regression analysis predicting %BF using nutrient intake as the three macronutrients (model 2) was also statistically significant (*F* (7, 192) = 10.24, *P* < 0.01), explaining approximately 25 % of the variance (adjusted R^2^ = 0.25). Among the independent variables, PAL (standardized *β* = −0.38, *P* < 0.01), FI (standardized *β* = 0.26, *P* = 0.01), CI (standardized *β* = −0.25, *P* = 0.01) and breakfast consumption frequency (standardized *β* = −0.13, *P* = 0.03) were significantly associated with %BF.

The multiple regression analysis predicting AMM using model 1 was statistically significant (*F* (5, 194) = 13.13, *P* < 0.01), explaining approximately 23 % of the variance (adjusted R^2^ = 0.23). Among the independent variables, PAL (standardized *β* = 0.46, *P* < 0.01) and breakfast consumption frequency (standardized *β* = 0.15, *P* = 0.02) were significantly associated with AMM. The multiple regression analysis predicting AMM using model 2 was also statistically significant (*F* (7, 192) = 11.75, *P* < 0.01), explaining approximately 27 % of the variance (adjusted R^2^ = 0.27). Among the independent variables, PAL (standardized *β* = 0.40, *P* < 0.01), PI (standardized *β* = 0.32, *P* < 0.01), FI (standardized *β* = −0.31, *P* < 0.01) and breakfast consumption frequency (standardized *β* = 0.15, *P* = 0.02) were significantly associated with AMM.

## Discussion

This is the first study to examine the relationship between breakfast skipping and body composition in female university students with normal BMI. Although body weight and BMI were not significantly different between the two groups, % BF was significantly higher and FFM was significantly lower in the SKIP group than in the TAKE group, with moderate effects. The SKIP group had a significantly higher proportion of body fat of >30 % (55.8 %) than the TAKE group (34.1 %), indicating that individuals with NW-O are more likely to be found among students who do not eat breakfast every day. Multiple regression analyses revealed that %BF and AMM were negatively and positively associated with PAL and breakfast consumption frequency, respectively. These results suggest that eating breakfast is necessary to prevent NW-O. Previous studies also reported a significant positive association between breakfast frequency and muscle mass indices among university students [11,20]. An older individual who has obesity in addition to sarcopenia, a loss of muscle mass and muscle function, is considered to have “sarcopenic obesity” [31,32]. In the present study, AMM/BMI, which is used as an indicator of sarcopenic obesity and more strongly associated with age than muscle mass alone [33], was significantly lower in the SKIP group. This suggests that improving NW-O in female university students may be important for preventing sarcopenic obesity and lifestyle-related diseases due to NW-O in the future.

The SKIP group had a significantly lower MEQ score than the TAKE group in the present study, suggesting that the later chronotype is a potential factor for NW-O. There were significant differences in the percentages of MT, NT, and ET between the two groups; the percentage of ET individuals was significantly higher in the SKIP group than in the TAKE group. Approximately 40 % of participants in the SKIP group were ETs. Previous studies observed an association between skipping breakfast and later chronotypes or ET in university students [20,34]. One reason for the high prevalence of ET in the SKIP group may be the later time of waking up. Although the bedtime of ET individuals is later, those living in a morning-oriented society cannot delay their waking time because of classes or work on weekdays. Therefore, ET individuals experience sleep deficits on weekdays and sleep longer on weekends owing to a lack of social constraints, resulting in a greater difference in sleep duration between weekdays and weekends [35]. In particular, women may be at a higher risk of developing adverse health symptoms because they have a greater difference in sleep duration on weekdays than men [36]. Consequently, ET individuals may sleep until the last minute before work or class on weekday mornings and therefore may not have time to eat breakfast in many cases. In fact, a significant positive correlation between chronotype and wake time has been observed among university students [37]. This difference in chronotypes may account for the high number of ET individuals in the SKIP group. The results of the present study suggest that chronotype-specific interventions may be required to prevent NW-O and future sarcopenic obesity in female university students, particularly those in the ET group.

Previous studies investigating breakfast skipping and chronotypes in relation to muscle mass indices have focused on muscle mass or energy and protein intake [20,38]. Multiple regression analysis in this study also revealed significant associations between %BF and FI (positively) and CI (negatively) and between AMM and PI (positively) and FI (negatively), respectively. Therefore, a study should include these three macronutrients and examine students with a normal-range BMI in relation to NW-O. Our data showed that although no significant differences in EI were found between the TAKE and SKIP groups, the %FI was significantly higher in the SKIP group than in the TAKE group. Although the proportion of ET individuals in the present study was higher in the SKIP group than in the TAKE group, similar or smaller differences in EI according to the chronotype have been reported [24,39,40]. However, ET individuals have been reported to have a higher proportion of breakfast skipping and lower energy intake at breakfast than MT individuals, while also having a higher energy intake from dinner and snacks during the afternoon and night [39–41]. Previous studies have reported higher intakes of lipids, carbohydrates, sodium, and polyunsaturated fatty acids [41], or sucrose and saturated fatty acids [42] in ET individuals than in MT individuals. Furthermore, a later chronotype has also been reported to be associated with a lower overall dietary quality index [43]. Therefore, ET individuals have a poor overall dietary balance, and their high prevalence in the SKIP group in the present study may have led to the higher %FI. Nutritional status in this study could only be assessed in terms of total daily amounts. To clarify this, future research should examine and compare the nutrient intake of each of the three meals and snacks.

This study had some limitations. First, this was a cross-sectional study, and the causal relationships among breakfast skipping, chronotype, and body composition are unclear. Therefore, the mechanism of the relationship between skipping breakfast and body composition needs to be investigated in more detail in the future, including a longitudinal study. Second, although the sample size in this study was similar to that in previous studies examining the association between skipping breakfast and body composition in university students [20,37], it may have been inadequate to ensure the generalizability of our findings. Furthermore, given the specific issues related to NW-O in women, the present study only included female participants. However, it cannot be ruled out that fluctuations in the menstrual cycle may have influenced body composition measurements obtained using the BIA method, although some studies suggest that such effects are not substantial [44, 45]. Further research is required to investigate this association at more universities with a larger number of participants, including both male and female participants. Third, as described in the Discussion section, the dietary survey used in this study could only assess total daily intake. Therefore, this study could not determine whether ET individuals consumed more energy at dinner or snacks during the afternoon and night. PAL was also assessed using a questionnaire, and there is a need to use an objective method, such as an activity meter. Finally, the relationship between chronotypes and lifestyle should be evaluated separately on weekdays and weekends. For example, poor diet among ET individuals is likely to be more pronounced on weekends [39]. Previous studies on PAL have also reported longer sleep durations on weekends in ET than in MT, and longer sleep durations on weekends were associated with lower physical activity on weekends [35]. Disruption of circadian regulation (e.g. misalignment between sleep patterns between weekdays and weekends) can lead to obesity and heart disease [46]. If chronotypic differences in lifestyle are not readily apparent on weekdays owing to social constraints, there is a strong need to examine these differences separately on weekdays and weekends. Future studies should address these issues in detail.

## Conclusion

This study investigated the relationship between breakfast skipping and body composition, chronotype, and nutritional status in female university students with a normal BMI. Although body weight and BMI were not significantly different between the two groups, %BF was significantly higher, and FFM was significantly lower in the SKIP group than in the TAKE group. The SKIP group had a significantly higher proportion of body fat >30 % (55.8 %) than the TAKE group (34.1 %). Although the total energy intake was not significantly different between the two groups, the %FI was significantly higher in the SKIP group. The multiple regression analyses showed that %BF and AMM were negatively and positively associated with PAL and breakfast consumption frequency, respectively. The SKIP group had a significantly lower MEQ score and a significantly higher number of ET individuals (41.9 %) than the TAKE group (23.5 %). These results suggest that the habit of breakfast consumption and chronotype-specific lifestyle interventions are necessary to prevent NW-O.

## Figures and Tables

**Fig. 1:**
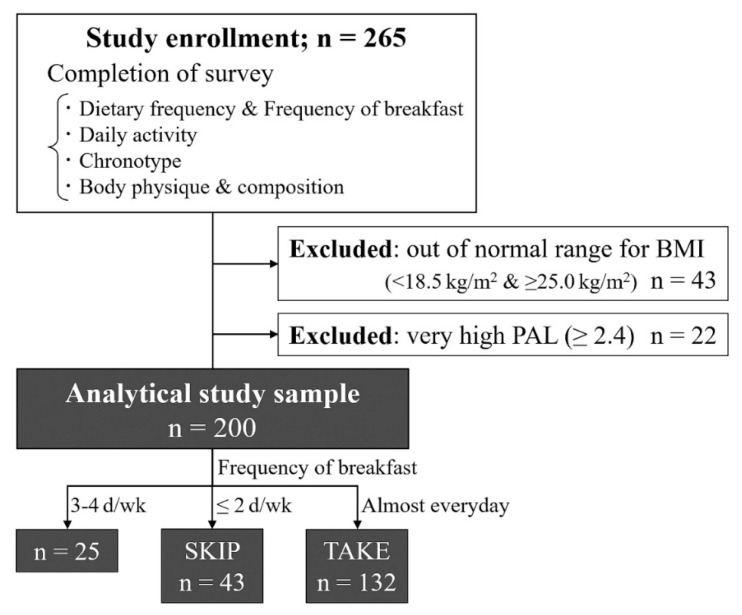
Flow diagram outlining participant selection for this study.

**Table 1:** Means and standard deviations of parameters for all participants and for each group

	ALL (n = 200)			SKIP group (n = 43)			TAKE group (n = 132)			SKIP vs. TAKE group*	Effect size
*Age (years)*	18.6	±	0.6	18.6	±	0.5	18.6	±	0.7	*P* =0.76	*d* = 0.09
*Height (cm)*	158.1	±	6.0	156.4	±	5.2	158.4	±	6.2	*P* = 0.09	*d* = 0.32
*Body weight (kg)*	53.1	±	5.9	52.4	±	5.5	53.5	±	6.1	*P* = 0.37	*d* = 0.17
*BMI (kg/m* *^2^* *)*	21.2	±	1.7	21.4	±	1.8	21.3	±	1.6	*P* = 0.70	*d* = 0.08
***%BF (%)***	**28.2**	**±**	**4.6**	**30.0**	**±**	**4.1**	**28.0**	**±**	**4.4**	***P*** ** = 0.01**	***d*** ** = 0.47**
***FFM (kg)***	**39.1**	**±**	**3.9**	**37.7**	**±**	**3.7**	**39.4**	**±**	**3.9**	***P*** ** = 0.01**	***d*** ** = 0.44**
***AMM (kg)***	**16.96**	**±**	**2.36**	**16.20**	**±**	**2.03**	**17.16**	**±**	**2.41**	***P*** ** = 0.05**	***d*** ** = 0.41**
***AMM/BMI (kg/kg/m*** *^2^* ***)***	**0.80**	**±**	**0.11**	**0.76**	**±**	**0.09**	**0.81**	**±**	**0.11**	***P*** ** = 0.01**	***d*** ** = 0.48**
***MEQ score***	**46.3**	**±**	**8.9**	**42.5**	**±**	**8.8**	**47.8**	**±**	**8.3**	***P*** ** < 0.01**	***d*** ** = 0.63**
*PAL*	1.78	±	0.30	1.79	±	0.31	1.78	±	0.29	*P* = 0.90	*d* = 0.03
*EI (kcal)*	1730.9	±	446.1	1739.5	±	520.0	1728.3	±	395.0	*P* = 0.53	*d* = 0.03
*PI (g)*	61.9	±	17.9	63.2	±	20.1	61.7	±	17.0	*P* = 0.79	*d* = 0.08
*% PI (%)*	13.5	±	1.6	13.2	±	1.6	13.6	±	1.7	*P* = 0.11	*d* = 0.25
*FI (g)*	59.3	±	19.5	60.9	±	23.7	58.9	±	17.8	*P* = 0.76	*d* = 0.10
***% FI (%)***	**29.8**	**±**	**4.3**	**30.8**	**±**	**4.3**	**29.4**	**±**	**4.4**	***P*** ** = 0.03**	***d*** ** = 0.32**
*CI (g)*	236.3	±	58.3	232.3	±	67.5	237.4	±	50.4	*P* = 0.07	*d* = 0.09
*% CI (%)*	56.8	±	4.6	56.0	±	4.7	57.0	±	4.7	*P* = 0.20	*d* = 0.21

Values are expressed as the mean ± standard deviation.

* Mann-Whitney *U* test.

BMI: body mass index; %BF: percentage of body fat; FFM: fat-free mass; AMM: appendicular muscle mass; MEQ: morningness-eveningness questionnaire; PAL: physical activity level; EI: intake of energy; PI: intake of protein; FI: intake of fat; CI: intake of carbohydrates; %PI: percentage of protein intake; %FI: percentage of fat intake; %CI: percentage of carbohydrates intake.

**Table 2:** Number of participants with body fat <30.0 % and ≥30.0 % among those with BMI between 18.5 and 24.9 kg/m^2^ in each group

	% BF (%)	
	< 30.0	≥ 30.0
*SKIP group*	19▼ (44.2 %)	24▲ (55.8 %)
*TAKE group*	87▲ (65.9 %)	45▼ (34.1 %)

χ^2^(1) = 5.53, *P* = 0.01; ▲ and ▼ indicate significantly higher and lower values (residual analysis). %BF: percentage of body fat.

**Table 3:** Number of participants of morning type (MT), neither type (NT), and evening type (ET) in each group

	Chronotype		
	MT	NT	ET
*SKIP group*	1 (2.3 %)	24 (55.8 %)	18▲ (41.9 %)
*TAKE group*	15 (11.3 %)	86 (65.2 %)	31▼ (23.5 %)

χ^2^(2) = 7.26, *P* = 0.03; ▲ and ▼ indicate significantly higher and lower (residual analysis).

**Table 4:** Results of the multiple regression analysis (n = 200)

Dependent variable: %BF							
Model 1				Model 2			
Variable	β	std β (95 % CI)	P value	Variable	β	std β (95 % CI)	P value
	
*Age*	−0.88	−0.12 (−0.24~0.00)	0.06	*Age*	−0.81	−0.11 (−0.24~0.01)	0.07
*PAL*	−6.53	−0.42 (−0.55~−0.30)	< 0.01	*PAL*	−5.97	−0.38 (−0.51~−0.26)	< 0.01
*MEQ*	−0.01	−0.02 (−0.15~0.10)	0.73	*MEQ*	−0.01	−0.02 (−0.15~0.10)	0.76
*EI*	0.00	−0.01 (−0.13~0.12)	0.91	*PI*	−0.01	−0.03 (−0.24~0.18)	0.77
*Breakfast*	−0.68	−0.14 (−0.28~−0.03)	0.02	*FI*	0.06	0.26 (0.05~0.06)	0.01
				
R^2^ = 0.24, adj R^2^ = 0.22, *F* = 12.04, *P* < 0.01				*CI*	−0.02	−0.25 (−0.46~−0.06)	0.01
				*Breakfast*	−0.58	−0.13 (−0.26~−0.01)	0.03
				
				R^2^ = 0.27, adj R^2^ = 0.25, *F* = 10.24, *P* < 0.01			

**Dependent variable: AMM**							
**Model 1**				**Model 2**			
**Variable**	**β**	**std β (95 % CI)**	**P value**	**Variable**	**β**	**std β (95 % CI)**	**P value**
	
*Age*	−0.26	0.07 (−0.05~0.19)	0.27	*Age*	0.22	0.06 (−0.06~0.018)	0.24
*PAL*	3.67	0.46 (0.34~0.58)	< 0.01	*PAL*	3.21	0.40 (0.28~0.52)	< 0.01
*MEQ*	0.01	0.01 (−0.12~0.13)	0.92	*MEQ*	−0.01	−0.01 (−0.13~0.12)	0.92
*EI*	0.00	0.00 (−0.12~0.12)	0.91	*PI*	0.04	0.32 (0.12~0.15)	< 0.01
*Breakfast*	0.35	0.15 (0.02~0.28)	0.02	*FI*	−0.04	−0.31 (−0.52~−0.12)	< 0.01
				
R^2^ = 0.25, adj R^2^ = 0.23, *F* = 13.13, *P* < 0.01				*CI*	0.01	0.02 (−0.16~0.23)	0.75
				*Breakfast*	0.34	0.15 (0.03~0.27)	0.02
				
				R^2^ = 0.30, adj R^2^ = 0.27, *F* = 11.75, *P* < 0.01			

std: standardized; %BF: percentage of body fat; PAL: physical activity level; MEQ: morningness-eveningness questionnaire; EI: intake of energy; PI: intake of protein; FI: intake of fat; CI: intake of carbohydrates; Breakfast: breakfast consumption frequency (four levels; 1 = Never, 2 = Rarely, 3 = Some days, and 4 = Every day); FFM: fat-free mass; AMM: appendicular muscle mass; adj: adjusted
